# On the Necessity of Practical Instruction in Medical Jurisprudence

**Published:** 1841-07

**Authors:** 


### Art. XII.

*De la Nécessité des Etudes Pratiques en Médecine Légale, &c.* Par H. L. Bayard, Docteur en Médecine, &c.—*Paris*, 1840. 8vo, pp. 30.
*On the Necessity of Practical Instruction in Medical Jurisprudence.* By Dr. Bayard.—*Paris*, 1840.

Dr. Bayard's attention has been called to the defective method of instruction in medical jurisprudence adopted in France, by the evidence given on the recent remarkable trials of Peytel and Lafarge. He has here published his reflections in a small pamphlet. We are induced to notice his essay because if, as he alleges, his remarks apply so strongly to the system of instruction followed in the French medical schools, they apply with much greater force to that adopted in our own.

In the author's opinion at least two thirds of the medical profession in France are compelled to execute the duties of medical witnesses, not from their want of professional knowledge, but from that knowledge never having been properly directed to the acquisition of medico-legal facts. In France, as in England, legal medicine is made a subject of purely *theoretical* instruction for a short period towards the close of a pupil's studies. The science has not, like the other branches taught at the Faculty, its corresponding *practical* course. Anatomy, midwifery, chemistry, and botany have their practical systems of instruction, but medical jurisprudence is without this important auxiliary.

Where instruction is thus purely theoretical the professor, however accomplished, can do little more than point out the course of study to his pupils, and quote to them the contradictory opinions of authors on different subjects. In treating of hanging and drowning, for example, he is confined to a mere verbal description—it is out of his power to procure subjects for illustration.

> "The *practical* knowledge acquired by the pupils is thus confined to a few notes taken during the course, or to the reading of some treatise on the subject. It is with this kind of preparation that they undergo a nominal examination." (p. 9.)

The young practitioner thus left to himself happens to be present at an accident, and he is required to make a medical report on the state of the wounded man. He finds himself here entirely at a loss: he may have arrested the hemorrhage and dressed the wound, but he has probably omitted to notice many minute points connected with the injury which are absolutely necessary in drawing up a report. Again, where a person is found dead from wounds, nothing but practical medico-legal knowledge can prevent a surgeon from falling into numerous errors, which may ultimately have the effect of defeating the ends of justice.

The author properly observes that it is not merely defective instruction which leads to such bad results, but the law itself throws obstacles in the way of those who are desirous of perfecting their knowledge of the subject.

Criminal investigations involving medico-legal questions are conducted in strict privacy. No one is allowed to be present except one or two judicial authorities and the medical inspectors regularly appointed. Thus a vast source of information is cut off from the profession; and in any similar case those who are thus excluded are incapable of acting with the requisite attention to legal forms. Dr. Bayard considers also that the poor remuneration made to medical witnesses in France is one cause of the indifference with which the subject is treated. Again, those who rank high in the profession as physicians or surgeons consider themselves thereby qualified to act as medical jurists. By them—and how many are there in our own country who indulge in this idea?—the study of the subject is regarded as superfluous; for they are satisfied with being masters of the sciences on which it is based. We quite agree with the author, and there are on record in both countries many trials which will bear out his assertion, that a man may be a very accomplished physician or surgeon, and at the same time but a very indifferent medical jurist.

So long as coroner's inquests are conducted in the imperfect manner in which they are at present, and so long as the subjects on which they are held are either not inspected at all, or inspected by one incompetent to the duty, medical jurisprudence must remain in England a purely *theoretical* study. The coroner is an officer not chosen by any test of ability, or by reference to his knowledge either of medicine or law; he has a direct interest in holding as many inquests as possible, so that many are held unnecessarily, others slovenly conducted; and in dispensing with post-mortem examinations, if a lawyer, he does not perceive the necessity for detaining the jury by such a form, and if a medical practitioner, he is apt to think his own knowledge sufficient: thus assuming the character of judge and witness. It would be difficult to say how society is benefited by such a system. The greater number of well-informed lawyers look with contempt on the coroner's court, and think if it were suppressed to-morrow society would be none the worse. There are few cases of importance requiring a coroner's interference which do not also come before a magistrate, and the investigation is here conducted in a clear and satisfactory manner.

Until coroners are selected upon other principles than at present, and until post-mortem inspections of all subjects of inquisitions are as much as possible thrown open for the benefit of science, there can be no hope of medical jurisprudence flourishing *practically* in England, nor can it be expected that sound medical evidence should be forthcoming when it is called for by the authorities of the law.

In France it is customary to appoint a certain number of physicians to each court, who act as referees in medico-legal investigations, and take the name of "*experts.*" The author finds fault with this arrangement, because there is no guarantee for these "experts" being well-informed in legal medicine. From what we know of English practice, however, we should consider an arrangement of this kind far superior to that which is now in use in this country, since among those chosen it is likely that at least some would have the ability to act. In England, under the coroner's inquest, the selection of a medical practitioner for conducting a medico-legal enquiry appears to rest in general with the beadle of a parish! and the rule followed by this functionary is commonly that of selecting the medical man who happens to live ***nearest*** to the spot where a dead body may be found. We apprehend M. Bayard would find much more reason to be dissatisfied with the English system than with that to which he objects.

In the French provinces, according to the author, the state of ignorance on these matters is much greater than in the capital. Mistakes are frequently occurring which can only be removed by an appeal to the more experienced medico-legal authorities of the capital. In proof of this view the Peytel case is brought forward, but not in our judgment very appropriately. The medical evidence here established that the prisoner murdered his wife by shooting her. Of this there could be but little doubt; but the evidence is objected to because the chest of the deceased was not properly examined for the marks of drowning, the prisoner having alleged in defence that he found his wife lying dead, with her face in water, although not entirely submerged. This is charged as an offence of omission against the medical witnesses, but their evidence, corroborated as it was by circumstances, brought the crime of murder clearly home to the prisoner, and we do not therefore see that the author has any ground to cavil, *ex post facto*, at the omission of what was really a subservient point in the enquiry.

Then comes the well-known case of Lafarge, which the author very properly calls the mysterious *drama* of Glandier. The evidence of the provincial witnesses is objected to, because before the analysis they did not seal up the vessels containing the suspected poisonous liquids, but as they afterwards discovered no poison, the objection is more technical than real. They are then condemned for presumptuous vanity in having framed a negative opinion when they had operated only on a portion of the liquids; the answer to which is, that although they might have gone further, there is no reasonable ground to suppose that by the *ordinarily recognized processes* for detecting arsenic with judicial certainty, they would have discovered any traces of that poison. The author looks upon Orfila as having vindicated the dignity of medical science from the disgrace thus brought upon it; but we must take leave to say, although we agree that the prisoner was properly convicted and punished for the crime, that we should be very sorry to see the chemical evidence relied on as a precedent in future trials. Unless arsenic be more unequivocally detected in a dead body than in our judgment it was in this case, the dignity of science will be best consulted by a witness declaring that there was insufficient chemical evidence of the presence of poison. Nevertheless, all who have read the proceedings of this singular case, including the account of the decoction of the body of the deceased in the open air, and before the court in which the trial was going on, must think with the author that it was truly a mysterious drama. Although M. Bayard may be right in his opinion as to the deficiencies of the French provincial practitioners, we think he has not been happy in his selection of illustrations to support it.

